# Work challenges and cancer-related cognitive impairment: level of education accounts for unmet needs beyond cognitive impairment severity

**DOI:** 10.1007/s00520-026-10420-8

**Published:** 2026-02-12

**Authors:** Darren Haywood, Susan L. Rossell, Ashley Henneghan, Frank D. Baughman, Jennifer Haywood, Evan Dauer, Annika Hegde, Ahmed A. Moustafa, Nicolas H. Hart

**Affiliations:** 1 Human Performance Research Centre, INSIGHT Research Institute, Faculty of Health, University of Technology Sydney (UTS), Sydney, NSW Australia; 2 Department of Mental Health, St. Vincent’s Hospital Melbourne, Fitzroy, VIC Australia; 3 Department of Psychiatry, Faculty of Medicine, Dentistry and Health Sciences, University of Melbourne, Melbourne, VIC Australia; 4 School of Population Health, Faculty of Health Sciences, Curtin University, Bentley, WA Australia; 5 Centre for Mental Health and Brain Sciences, Swinburne University of Technology, Hawthorn, VIC Australia; 6 School of Nursing, University of Texas at Austin, Austin, TX USA; 7 Department of Oncology, Dell Medical School, The University of Texas at Austin, Austin, TX USA; 8 Centre for Data Analytics & School of Psychology, Bond University, Gold Coast, QLD Australia; 9 Caring Futures Institute, College of Nursing and Health Sciences, Flinders University, Adelaide, SA Australia; 10 Cancer and Palliative Care Outcomes Centre, Faculty of Health, Queensland University of Technology (QUT), Brisbane, QLD Australia; 11 Exercise Medicine Research Institute, School of Medical and Health Science, Edith Cowan University, Joondalup, WA Australia; 12 Institute for Health Research, University of Notre Dame Australia, Fremantle, WA Australia; 13 Department of Human Anatomy and Physiology, the Faculty of Health Sciences, University of Johannesburg, Johannesburg, South Africa

**Keywords:** Cancer-related cognitive impairment, CRCI, Work, Occupational functioning, Return to work, Cancer

## Abstract

**Purpose:**

Cancer-related cognitive impairment (CRCI) can impact cancer survivors’ return to work and occupational functioning for many years following the completion of cancer treatment. Understanding the characteristics of CRCI-related occupational difficulties and unmet supportive care needs is critical for the development of effective interventions. Within occupational roles, a higher level of education is typically associated with greater cognitive load and different demands. The objective of this study was to examine whether the level of education accounted for CRCI-related (a) occupational difficulties and (b) occupational unmet supportive care needs, beyond the contributions of perceived cognitive functioning and employment level.

**Methods:**

A cross-sectional design was utilised using a sub-section of an existing self-reported dataset involving 358 employed cancer survivors, comprising demographic and clinical data, as well as data from MASCC COG-IMPACT and PROMIS Cog tools. Hierarchical multiple linear regression models were used for hypothesis testing.

**Results:**

After accounting for perceived cognitive functioning and employment level, highest level of education did not account for a significant amount of unique variance in CRCI-related occupational difficulties (*R*^2^ Change = 0.004, *F* Change _(1, 354)_ = 3.26, *p* = .147). However, after accounting for perceived cognitive functioning and employment level, highest level of education did account for a significant amount of unique variance in CRCI-related occupational unmet needs (*R*^2^ Change = 0.011, *F* Change _(1, 354)_ = 4.75, *p* = .030).

**Conclusion:**

Theoretical explanations of findings, including compensatory accommodations such as role adjustment and differences in the availability of occupational support, are provided. It is proposed that those with higher levels of education may not have access to appropriate levels of supportive care as it relates to CRCI in occupational settings, thus potentially informing future interventions. It is proposed that individuals with higher levels of education may experience gaps in supportive care for CRCI, particularly in relation to maintaining work performance and meeting occupational expectations. This suggests that future interventions should consider strategies to better support cognitive functioning in workplace contexts for cancer survivors.

## Introduction

Cancer-related cognitive impairment (CRCI) is characterised by a decline in cognitive functioning (including memory, concentration, attention, inhibition, and speed of processing) as a result of cancer, its treatments, and their impacts on psychological well-being [1, 2]. CRCI can impact cancer survivors long-term, with CRCI reported even decades after the end of cancer treatment, including when cancer-free [1, 3–6]. Although most cancer diagnoses occur in older adults, improvements in early detection and treatments, as well as an increasing global retirement age, mean that an increasing number of people are living beyond cancer during their core working years [7, 8]. CRCI has been shown to significantly impact the occupational functioning of people living beyond cancer [9–12]. This includes the return-to-work process following cancer as well as the ability to perform work-specific tasks and responsibilities of their occupation relative to their previous standard. Occupational functioning for cancer survivors has significant implications for psychosocial as well as financial well-being, with greater occupational difficulties and unmet needs related to CRCI significantly associated with greater psychosocial distress, additional financial concerns, and overall poorer quality of life [9–12].

Occupational functioning is central to self-identity and psychosocial well-being, particularly for those with higher levels of education, due to the additional time and financial commitment required toward achieving the level of education that typically aligns with their job role [13, 14]. Those with higher levels of education also tend to have occupations with greater cognitive demands and fewer physical demands [15, 16]. While exceptions exist, such as taxi drivers or electricians, whose roles demand high physical and cognitive performance but not necessarily advanced education, it can generally be suggested that cancer survivors who have lower levels of education often work in more physically demanding jobs. For these individuals, physical side effects of cancer, such as physical fatigue and pain, are likely to play a central role in their occupational functioning. Indeed, research shows that those with more physically demanding occupations living with and beyond cancer return to work at a slower rate compared to those in less physically demanding roles [17]. Conversely, this suggests that for cancer survivors who experience CRCI with higher levels of education, and thus typically with more cognitively demanding roles, they may experience more significant *CRCI-related* occupational difficulties.

People living with and beyond cancer who have a greater severity of CRCI experience more significant occupational challenges [18, 19]. Further, it is common for those experiencing long-term effects of cancer and its treatment, such as CRCI, to return or adjust to working in a reduced full-time equivalency to manage occupational demands [18, 19]. However, it is important to understand the unique impacts level of education may have on CRCI-related occupational difficulties and unmet needs to inform screening, assessment, and the development and implementation of tailored interventions. It is understood that level of education is not only associated with job roles with differing cognitive demands, but also levels and types of responsibilities, consequences of errors, level of autonomy, and available job role supports [13–15]. It is therefore possible that, beyond just the impact of CRCI severity and employment level (i.e. part-time, full-time, etc.), a higher level of education may result in additional CRCI-related difficulties and unmet supportive care needs. This may be through mechanisms such as additional cognitive demand of job roles, additional occupational responsibilities (i.e. staff reporting to the person living with and beyond cancer, financial handling, etc.), and the potential for lesser available job role support due to specific knowledge and skillset requirements. Individuals with higher levels of education may be more aware of available supportive care options and the standards associated with such care. This heightened awareness could lead to greater perception of unmet needs related to CRCI. Conversely, because higher education is often associated with greater income, cancer survivors may have an increased ability to access additional supportive care when compared to individuals with lower education levels, resulting in lesser unmet needs related to CRCI. However, to date, no research has been able to examine the relation between level of education and both CRCI-related difficulties and unmet needs, after accounting for cognitive functioning, due to the lack of a suitable assessment tool. Recently, the MASCC COG-IMPACT [18, 20], the first purpose-built unmet needs assessment tool describing the personal impact of CRCI, was developed and validated. As the MASCC COG-IMPACT [18, 20] includes an occupational/vocational function subscale measured across two indices, difficulties and unmet needs, it offers the ideal platform to examine the relation between level of education and both CRCI-related occupational difficulties and unmet needs.

The aim of the study was to examine if the level of education could account for CRCI-related (a) occupational difficulties and (b) occupational unmet supportive care needs after accounting for the contributions of perceived cognitive functioning and employment level. Accordingly, this study had two hypotheses: (1) after accounting for perceived cognitive functioning and employment level, a higher level of education would significantly account for greater levels of work-related difficulties resulting from CRCI, and (2) after accounting for perceived cognitive functioning and employment level, a higher level of education would significantly account for greater levels of work-related unmet needs resulting from CRCI.

## Methods

### Design

A cross-sectional design utilising participant self-reports was employed. Ethics clearance was granted by the St. Vincent’s Hospital Melbourne Human Research Ethics Committee (PID05582), and the research was conducted in accordance with the Declaration of Helsinki. All participants provided informed consent. This study used an existing dataset from the development and validation of the MASCC COG-IMPACT [18]. Additional details of the design and procedure relating to the collected data can be found in the published protocol [21].

### Participants

Eligible participants were adults (18+ years) with a previous cancer diagnosis, who had successfully completed curative-intent treatment, and did not currently have cancer (i.e. no known cancer recurrence). All participants self-identified as experiencing CRCI and being fluent in English (reading and speaking). For this study, we analysed data from participants within an existing dataset who reported that they were currently employed. The sole exclusion criterion was a self-reported diagnosis of another neurocognitive or neurological condition.

Recruitment focused on individuals who identified as experiencing CRCI (i.e. self-identified as experiencing memory, attention, concentration, or other cognitive challenges that they attribute to their cancer, as detailed in the survey invitation)—rather than solely those meeting predetermined quantitative neuropsychological thresholds—recognising the qualitative significance of lived experiences of cognitive changes post-diagnosis in shaping unmet supportive care needs.

### Recruitment

Participants were recruited through Prolific [22], a well-regarded platform recognised for its reliability and validity in research participant sourcing. It is widely utilised in neuropsychological studies, including those within oncology contexts [9, 18, 23]. The survey invitation was visible to all of those who reported a cancer diagnosis and included the detailed inclusion and exclusion criteria, including the requirement of perceiving to experience CRCI.

### Measures

A portion of the existing dataset from the development and validation of the MASCC COG-IMPACT was used [21]. The self-report survey included demographic and clinical questions, including employment level (coded as 1 = casual, 2 = part-time, 3 = full-time) and level of education (coded as 1 = primary school, 2 = secondary school, 3 = vocation, 4 = bachelor’s degree, 5 = master’s degree, 6 = PhD/MD), as well as the MASCC COG-IMPACT [18], and Patient-Reported Outcomes Measurement Information System–Cognitive Function Scale 8a (PROMIS Cog) [24].

The MASCC COG-IMPACT is a 55-item and 8-subscale validated measure of CRCI-related unmet needs [18]. A subsequent CRCI-related ‘Difficulties’ and ‘Unmet Needs’ score can be calculated for each MASCC COG-IMPACT subscale (see [18]). For this study, the ‘Occupational/Vocational Functioning’ subscale was used. CRCI-related occupational functioning difficulties are defined as difficulties feeling and being prepared and capable of working at their own expected capacity and capability, as well as difficulties related to others’ perceptions of their capacity and capability due to CRCI. CRCI-related occupational functioning unmet supportive care needs are defined as needing help or support regarding the CRCI-related occupational difficulties. The MASCC COG-IMPACT measures CRCI-related difficulties and associated unmet supportive care needs over the previous month, and higher scores reflect greater difficulties and unmet needs. If a CRCI-related difficulty is endorsed by a participant, they are then asked to indicate their level of unmet supportive care needs related to that specific difficulty. The Difficulties indices are responded to via a ‘no’ or ‘yes’ indication, and the Unmet Needs indices are responded to by endorsing one of the following response options: ‘I do not need any additional support’, ‘my need for support is satisfied’, ‘I have a low need for additional support’, ‘I have a moderate need for additional support’, or ‘I have a high need for additional support’. An example item from the Occupational/Vocational Functioning subscale is ‘Because of my CRCI, I have difficulty understanding complex ideas, concepts, or processes at work/volunteering/school’. Subscale scores for the Difficulties indices range from 0 to 1, while the subscale scores for the Unmet Needs indices range from 0 to 3. Higher scores reflect greater difficulties and unmet needs, respectively (see Haywood et al. [18] for details on the scoring procedures). The PROMIS Cog [24] is a highly validated self-report assessment of cognitive function for cancer survivors recommended by the Neuroscience Initiative Working Group for use in CRCI research [25, 26]. The PROMIS Cog provides an overall total score across its items, which is measured relating to the previous month, with greater scores reflecting greater cognitive functioning. The raw total score was utilized in all analyses.

### Analysis

To characterise the sample, the study employed descriptive statistics and frequency analyses. Correlational methods were then applied to address the primary research objectives. Specifically, means, standard deviations, counts, ranges, and percentages are used to detail the sample’s characteristics, and Pearson’s bivariate correlation analyses (for continuous variables and ordinal variables) and point-biserial correlation analyses (for binary variables) were used to explore relationships between the variables. The strength of effect for the bivariate correlations was interpreted as follows: small *r* ≤ 0.3, medium *r* = 0.31 to *r* = 0.69, large *r* ≤ 0.70 [21]. Two-step multiple regression analyses were then used to test the hypotheses. The predictor variables for each multiple regression analysis were the same: PROMIS Cog total score and employment level entered on step one, and level of education entered on step two. In line with each hypothesis, the first multiple regression analysis had the dependent variable of the MASCC COG-IMPACT Occupational/Vocational ‘difficulties’ subscale score, and the second multiple regression analysis had the dependent variable of the MASCC COG-IMPACT Occupational/Vocational ‘unmet needs’ subscale score. An alpha level of 0.05 was used for all analyses to determine statistical significance. Statistical Package for the Social Sciences (SPSS) version 30 was used for all analyses.

## Results

### Participants

A total sample of 358 cancer survivors out of the 491 in the full dataset [18] was used for this study, as 138 participants reported not to be in employment. Sample characteristics are reported in Table 1.

**Table 1:** Sample characteristics

Characteristic	Mean (SD)/count (*N* = 358)
Age (years)	*M* = 41.9 years (SD = 13.1)
Sex at birth	
Male	98 (27.4%)
Female	260 (72.6%)
Ethnicity	
Caucasian	190 (53.1%)
African/African American	129 (36.0%)
Asian	13 (3.6%)
Hispanic or Latino	10 (2.8%)
Native American/American Indian	2 (0.6%)
Other	14 (3.9%)
Employment level	
Full-time	285 (79.6%)
Part-time	67 (18.7%)
Casual	6 (1.7%)
Highest level of education	
PhD/MD	26 (7.3%)
Master’s degree	89 (24.9%)
Bachelor’s degree	147 (41.1%)
Vocation (technical school, certificate)	50 (14.0%)
Secondary school (high school)	46 (12.8%)
Primary cancer type	
Breast	121 (33.8%)
Prostate	15 (4.2%)
Bowel/colorectal	22 (6.1%)
Melanoma	13 (3.6%)
Lung	16 (4.5%)
Lymphoma	29 (8.1%)
Leukaemia	20 (5.6%)
Brain	14 (3.9%)
Pancreatic	3 (0.8%)
Cervical	16 (4.5%)
Thyroid	22 (6.1%)
Testicular	15 (4.2%)
Uterine	9 (2.5%)
Ovarian	16 (4.5%)
Sarcoma	3 (0.8%)
Kidney	5 (1.4%)
Bladder	1 (0.3%)
Other	18 (5.0%)
Country of residence	
United States of America	122 (34.1%)
United Kingdom	73 (20.4%)
South Africa	100 (27.9%)
Others	63 (17.6%)
Treatments received	
Chemotherapy	253 (70.7%)
Radiation	184 (51.4%)
Hormone treatment	103 (28.8%)
Targeted therapies	54 (15.1%)
Surgery	75 (20.9%)
Immunotherapy	68 (19.0%)
Other	7 (2.0%)
Years since diagnosis	*M* = 11.1 years (SD = 11.3)

Rounding and multiple treatment modalities may result in percentages not equalling 100%

### Correlation analysis

Exploratory bivariate correlations between all utilized variables were conducted to examine relationships between the variables.

The associations are presented in Table 2. CRCI-related occupational difficulties were significantly positively associated with CRCI-related occupational unmet needs (*r* = 0.66, *p* < 0.01) and negatively associated with cognitive functioning (*r* = −0.54, *p* < 0.01), with medium associations, as well as negatively associated with employment level (*r* = 0.18, *p* < 0.01), and positively associated with cancer stage at most progressed (*r* = 0.14, *p* < 0.05), with small associations. CRCI-related occupational unmet needs were also significantly negatively associated with cognition (*r* = −0.44, *p* < 0.01), with a medium association, as well as positively associated with cancer stage at most progressed (*r* = 0.13, *p* < 0.05), with a small association. In addition to being positively associated with the MASCC COG-IMPACT indices, cognition was significantly negatively associated with sex (*r* = −0.17, *p* < 0.01), cancer stage at initial diagnosis (*r* = −0.22, *p* < 0.01), and cancer stage at most progressed (*r* = −0.24, *p* < 0.01), all with small associations. Level of education was significantly negatively associated with age (*r* = −0.19, *p* < 0.01) and cancer stage at initial diagnosis (*r* = −0.12, *p* < 0.05), both with small associations. In addition to its negative association with CRCI-related occupational difficulties, employment level was significantly negatively associated with age (*r* = −0.18, *p* < 0.01) and significantly positively associated with cognitive functioning (*r* = 0.11, *p* < 0.05) and level of education (*r* = 0.17, *p* < 0.02), all with small associations.

**Table 2:** Bivariate correlations

	1	2	3	4	5	6	7	8	9
1. Age	–								
2. Sex	0.047	–							
3. Level of education	−0.192^**^	−0.082	–						
4. Employment level	−0.179^**^	−0.064	0.174^**^	–					
5. Days since diagnosis	0.199^**^	0.027	−0.039	−0.92	–				
6. Stage at initial diagnosis	0.183^**^	−0.006	−0.122^*^	−0.049	0.005	–			
7. Stage at most progressed	0.120^*^	0.001	−0.037	0.014	0.045	0.829^**^	–		
8. CRCI occupational difficulties	−0.033	−0.002	−0.004	−0.179^**^	−0.094	0.096	0.135^*^	–	
9. CRCI occupational unmet needs	−0.045	0.037	0.057	−0.085	−0.078	0.089	0.128^*^	0.664^**^	–
10. PROMIS Cog	−0.008	−0.168^**^	0.086	0.110^*^	0.096	−0.217^**^	−0.235^**^	−0.542^**^	−0.442^**^

^**^Correlation is significant at the 0.01 level (2-tailed)

^*^Correlation is significant at the 0.05 level (2-tailed)

Sex: 1 = male, 2 = female. *CRCI* cancer-related cognitive impairment

### CRCI-related occupational difficulties and unmet needs in relation to level of education

Table 3 displays the descriptive statistics of the MASCC COG-IMPACT Difficulties indices for the Occupational/Vocational Functioning subscale per level of education. No participant within the sample had their highest level of education be primary school.

**Table 3:** Difficulties indices descriptive statistics

Highest level of education	Mean	Median	Std. deviation	Minimum	Maximum
Secondary school	0.335	0.30	0.308	0.00	1.0
Vocation	0.488	0.55	0.310	0.00	1.0
Bachelor degree	0.506	0.50	0.312	0.00	1.0
Masters degree	0.403	0.30	0.343	0.00	1.0
PhD, MD	0.354	0.30	0.326	0.00	1.0
Total	0.445	0.40	0.325	0.00	1.0

*PhD* Doctor of Philosophy, *MD* Medical Doctorate

**Fig. 1:**
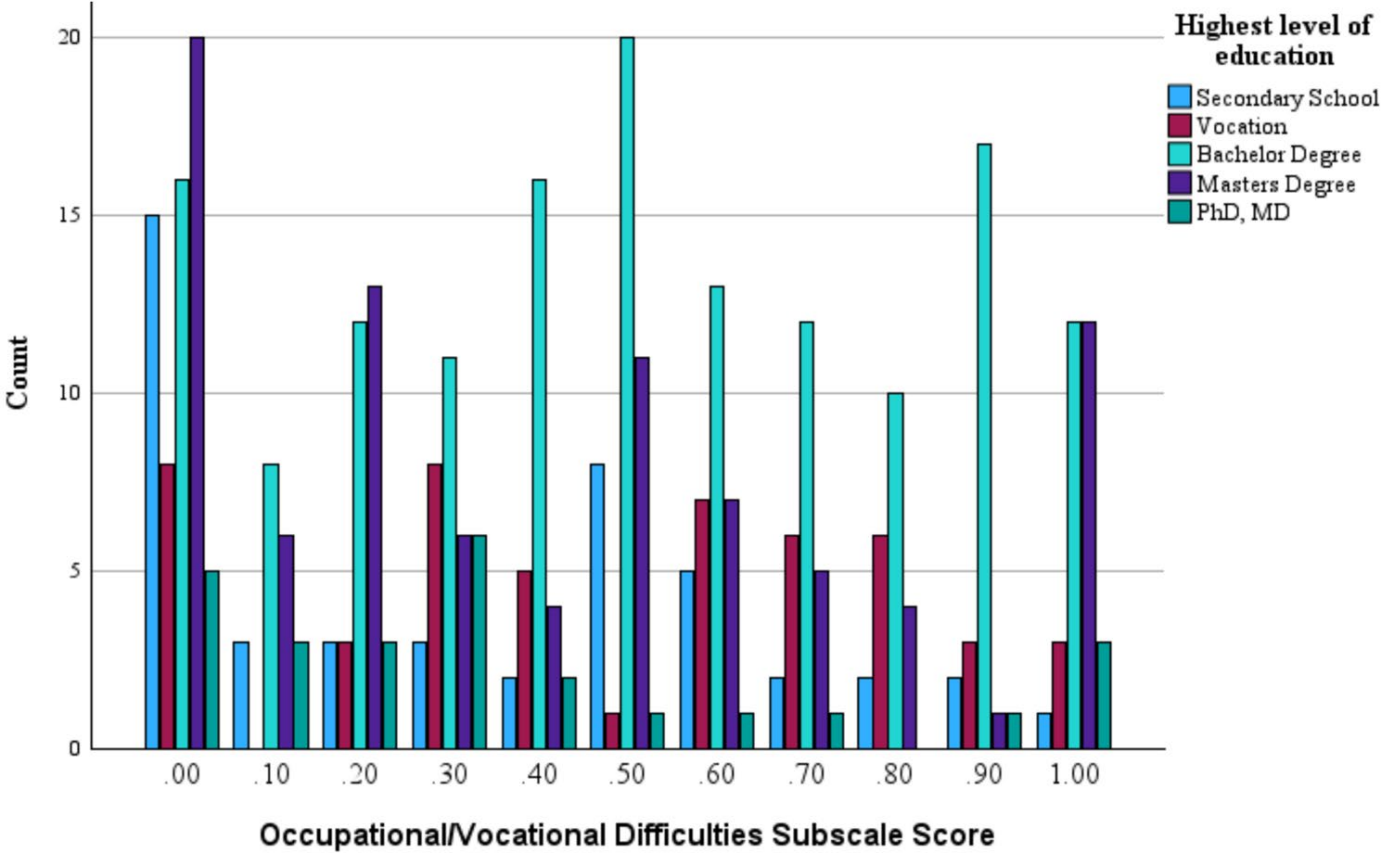
Difficulties indices scores as per highest level of education

Figure 1 depicts the distribution of Difficulties indices scores as per the highest level of education on the Occupational/Vocational Functioning subscale.

Table 4 displays the descriptive statistics of the MASCC COG-IMPACT Unmet Needs indices for the Occupational/Vocational Functioning subscale per level of education.

**Table 4:** Unmet needs indices descriptive statistics

Highest level of education	Mean	Median	Std. deviation	Minimum	Maximum
Secondary school	0.270	0.00	0.438	0.00	2.20
Vocation	0.398	0.10	0.699	0.00	2.60
Bachelor degree	0.535	0.30	0.635	0.00	2.80
Masters degree	0.405	0.10	0.650	0.00	2.80
PhD, MD	0.427	0.20	0.550	0.00	1.70
Total	0.442	0.20	0.624	0.00	2.80

**Fig. 2:**
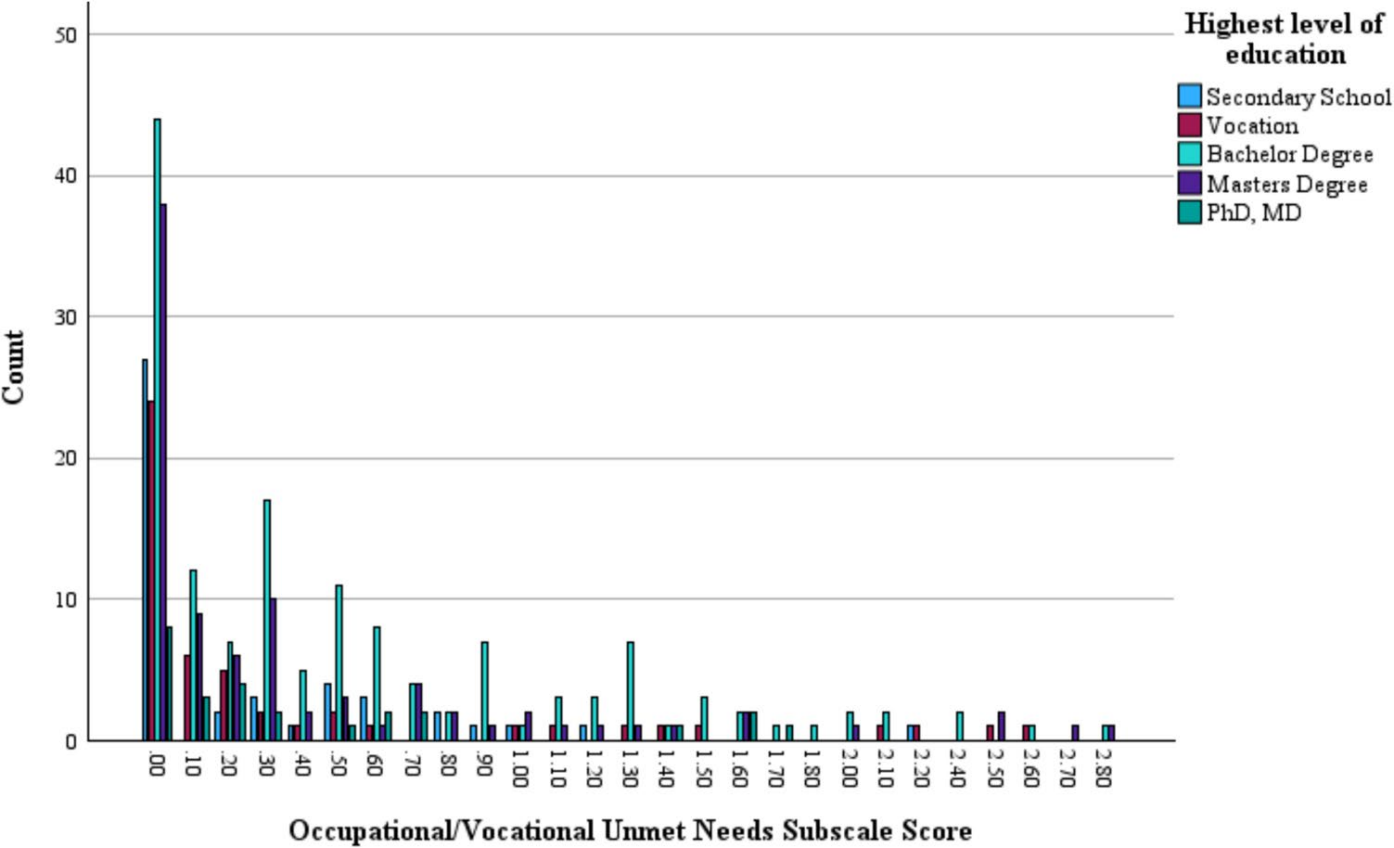
Unmet needs indices scores as per highest level of education

Figure 2 depicts the distribution of Difficulties indices scores as per the highest level of education on the Occupational–Vocational Functioning subscale.

### Multiple regression analyses

#### Hypothesis 1

The results of the multiple regression analysis are presented in Table 5.

**Table 5:** Multiple regression to test if level of education can account for CRCI-related difficulties

Model	Unstandardised coefficients		Standardised coefficients	*t*	Sig	Correlations			
	*B*	Std. error	Beta			95% CI	Zero-order	Partial	Part
*Occupational/vocational functioning CRCI-related difficulties*									
*Step 1*									
Cognitive functioning	−0.020	0.002	−0.529	−11.91	< 0.001	−0.023, −0.016	−0.542	−0.534	−0.526
Employment level	−0.081	0.032	−0.121	−2.72	0.007	−0.149, −0.024	−0.172	−0.143	−0.120
*Step 2*									
Cognitive functioning	−0.020	0.002	−0.533	−12.00	< 0.001	−0.023, −0.017	−0.542	−0.538	−0.529
Employment level	−0.094	0.032	−0.132	−2.93	0.004	−0.158, −0.031	−0.179	−0.154	−0.129
Level of education	0.019	0.013	0.065	1.46	0.147	0.046, −0.004	−0.004	0.077	0.064

On step 1, perceived cognitive functioning and employment level significantly accounted for 30.9% of variance in CRCI-related occupational difficulties (*R*^2^ = 0.309, *F*_(2, 355)_ = 79.20, *p* < 0.001). The addition of level of education on step 2 did not significantly improve the model’s predictive utility (*R*^2^ Change = 0.004, *F* Change _(1, 354)_ = 3.26, *p* = 0.147), with perceived cognitive functioning accounting for a significant 28.0% of unique variance in CRCI-related occupational difficulties, employment level accounting for a significant 1.7% of variance, and level of education accounting for a non-significant 0.4% of unique variance in CRCI-related occupational difficulties. Poorer perceived cognitive functioning and lower employment level were associated with greater CRCI-related occupational difficulties. Therefore, as level of education did not account for a significant amount of unique variance in CRCI-related occupational difficulties after accounting for perceived cognitive functioning, hypothesis 1 was not supported.

#### Hypothesis 2

The results of the multiple regression analysis are presented in Table 6.

**Table 6:** Multiple regression to test if level of education can account for CRCI-related difficulties

Model	Unstandardised coefficients			Standardised coefficients	*t*	Sig	Correlations						
	*B*	Std. error		Beta			95% CI	Zero-order		Partial		Part	
*Occupational/vocational functioning CRCI-related unmet needs*													
*Step 1*													
Cognitive functioning	−0.031		0.003	−0.438	−9.15	< 0.001	−0.038, −0.025		−0.442		−0.437		−0.435
Employment level	−0.051		0.066	−0.037	−0.771	0.441	−0.180, 0.079		−0.085		−0.041		−0.037
*Step 2*													
Cognitive functioning	−0.032		0.003	−0.445	−9.33	< 0.001	−0.039, −0.025		−0.442		−0.444		−0.441
Employment level	−0.075		0.066	−0.054	−1.23	0.261	−0.205, 0.056		−0.085		−0.060		−0.053
Level of education	0.060		0.027	0.105	2.18	0.030	0.006, 0.114		0.057		0.115		0.103

On step 1, perceived cognitive functioning and employment level significantly accounted for 19.7% of variance in CRCI-related occupational unmet needs (*R*^2^ = 0.197, *F*_(2, 355)_ = 49.47, *p* < 0.001). The addition of level of education on step 2 significantly improved the model’s predictive utility (*R*^2^ Change = 0.011, *F* Change _(1, 354)_ = 4.75, *p* = 0.030) with perceived cognitive functioning accounting for a significant 19.45% of unique variance in CRCI-related occupational unmet needs, employment level accounting for a non-significant 0.3% of unique variance, and level of education accounting for an additional significant 1.1% of unique variance in CRCI-related occupational unmet needs. Poorer perceived cognitive functioning and greater levels of education were associated with greater CRCI-related occupational difficulties. Therefore, as level of education accounted for a significant amount of unique variance in CRCI-related occupational unmet needs after accounting for perceived cognitive functioning, hypothesis 2 was supported.

## Discussion

This study examined if the level of education could account for CRCI-related (a) occupational difficulties and (b) occupational unmet supportive care needs after accounting for the contributions of perceived cognitive functioning and employment level. For people living beyond cancer, who perceived experiencing CRCI, higher levels of education did not significantly account for CRCI-related occupational difficulties; however, it did significantly account for CRCI-related occupational unmet needs.

Most CRCI research interfaced with occupational functioning focuses on both subjectively and objectively measured CRCI determination and/or severity and its relation to return-to-work, performance, and functioning at work [11, 12]. However, a significant gap exists in understanding the nuances of the relationship between CRCI and work, impacting the ability to develop tailored interventions. Our findings were consistent with the literature, as positive associations were found between perceived cognitive functioning and employment level, and negative associations between those variables and CRCI-related occupational unmet supportive care needs [9, 11, 12, 18]. However, contrary to expectations, higher levels of education were not associated with greater CRCI-related occupational difficulties beyond perceived cognitive functioning and employment level. While higher levels of education are typically associated with job roles of higher cognitive demands, this is not always the case. It is not uncommon for those with higher levels of education to have job roles of lower cognitive demand, including those outside of their educational disciplines, due to available employment opportunities [27]. The opposite is also true, with many roles requiring a lower level of education still having high cognitive demands [27]. However, across our sample, it was still expected, on balance, that an effect would be observed. One potential explanation is that many people living beyond cancer who return to work are provided compensatory measures such as role adjustment, including different roles or lightened workloads in the same full-time equivalency, and adjusted task responsibilities to account for the effects of cancer, including CRCI [28, 29]. While employment level was accounted for in the analyses, additional role adjustment in the context of CRCI may be particularly notable for those with higher education who, across the population, tend to have roles with greater cognitive demands. Therefore, role adjustments may have prevented additional CRCI-related occupational difficulties among those with higher education, even though they experienced greater unmet supportive care needs due to expectations of maintaining their previous responsibilities and capacity.

Beyond CRCI-related occupational difficulties, it is vital to understand CRCI-related unmet supportive care needs for those living beyond cancer, to ensure that required care to enhance functioning, wellbeing, and employment continuation is provided [20]. In line with expectations, higher levels of education were associated with greater CRCI-related occupational unmet needs beyond perceived cognitive functioning and employment level. While the lack of association between level of education and CRCI-related difficulties may be somewhat explained by role adjustment, the significant association between level of education and CRCI-related occupational unmet supportive care needs may reflect that for every difficulty faced, a disproportionately greater amount of associated unmet need results for those with higher education levels, emphasising that a greater level of supportive care intervention is required [28, 29]. Further, this may also reflect the availability of occupational supportive care in particular types of workplaces, particularly in the areas of responsibility and role adjustment, due to lesser availability of individuals with the required role knowledge and skillsets to substitute. Finally, those with higher levels of education may also be more acutely aware of available supportive care, and thus, if they are not receiving these to the level they deem required, they may report a greater level of unmet need. While it is important to acknowledge the relatively low proportion of unique variance level of education accounts for in CRCI-related occupational unmet supportive care needs, these findings, in corroboration with the body of literature, suggest a potentially interactive relationship between level of education, role adjustments, and unmet CRCI-related occupational supportive care needs for those in employment who are living beyond cancer.

### Strengths, limitations, and future research

This study had two key strengths. Firstly, the study drew on a substantial cohort of people living beyond cancer, including wide-ranging clinical and demographic profiles such as country of residence, age, gender, ethnicity, cancer types and stages, and treatment histories. Secondly, the study employed robust methodologies and statistical approaches, while using highly validated tools, thus enabling the generation of data-informed theoretical propositions.

This study also had limitations. Firstly, only those who completed curative intent treatment, were judged to be free of cancer (i.e. no cancer recurrence), and currently employed were included. While this allows the ability to draw stronger conclusions for this cohort, it retracts from the generalisability of findings to other important groups of cancer survivors, such as those undergoing treatment, those with metastatic cancer or advanced cancer, and those who have not yet returned to work due to CRCI. Future research should extend this assessment to these populations. Secondly, we did not incorporate data on occupational role or obtain data on role adjustment or career change. Thus, the theoretical interpretations of the potential mechanism underlying observed relationships could not be assessed and remain speculative. Future research should incorporate current and previous occupational role, as well as indicators of occupational adjustments and career changes. Thirdly, current employment level was used in analyses; however, change in employment level following cancer was not assessed. Future research should explore employment level change in this context. Fourthly, CRCI was not evaluated using objective assessments in this study, removing our ability to incorporate objective cognitive functioning in our analysis. Future research should incorporate objective cognitive assessment. Fifthly, we did not objectively measure cognitive and physical demands of job roles as reported by participants, mitigating our potential to explicitly explore their relation to CRCI difficulties and unmet needs. Sixthly, the large majority of participants had a higher level of education (73.3% with a Bachelor’s level degree or higher), and no participants had primary school as their highest level of education, impacting the applicability of the findings. Lastly, all variables, including clinical information, were obtained through self-reporting, which may introduce reporting inaccuracies. Future studies could consider incorporating medical records to enhance data reliability.

## Conclusion

Level of education significantly accounts for CRCI-related occupational unmet supportive care needs, but not CRCI-related occupational difficulties, after accounting for perceived cognitive functioning. People with higher levels of education who are living beyond cancer and perceive experiencing CRCI face additional CRCI-related occupational unmet supportive care needs relative to their CRCI-related occupational difficulties. This could be due to a lack of appropriate role adjustments, structural or functional job accommodations, or sufficient substitution of caretaker employees to cover roles in lieu of the CRCI-affected employee for people working in cognitively demanding job roles that may have additional responsibilities. These results provide key knowledge to inform assessment and interventions as well as future research.

## Data Availability

Anonymised data may be made available upon reasonable request of the corresponding author and to the satisfaction of the granting HREC.
